# The U1 antisense morpholino oligonucleotide (AMO) disrupts U1 snRNP structure to promote intronic PCPA modification of pre-mRNAs

**DOI:** 10.1016/j.jbc.2023.104854

**Published:** 2023-05-22

**Authors:** Qiumin Feng, Zejin Lin, Yanhui Deng, Yi Ran, Rui Yu, Andy Peng Xiang, Congting Ye, Chengguo Yao

**Affiliations:** 1 Center for Stem Cell Biology and Tissue Engineering, Key Laboratory for Stem Cells and Tissue Engineering, Ministry of Education, Sun Yat-Sen University, Guangzhou, Guangdong, China; 2 Key Laboratory of the Ministry of Education for Coastal and Wetland Ecosystems, College of the Environment and Ecology, Xiamen University, Xiamen, Fujian, China; 3 Department of Biochemistry, School of Medicine, Ningbo University, Ningbo, Zhejiang, China; 4 Advanced Medical Technology Center, The first Affiliated Hospital, Zhongshan School of Medicine, Sun Yat-sen University, Guangzhou, Guangdong, China; 5 Department of Genetics and Cell Biology, Zhongshan School of Medicine, Sun Yat-Sen University, Guangzhou, Guangdong, China

**Keywords:** U1 snRNP telescripting, intronic polyadenylation, premature cleavage and polyadenylation, premature transcription termination

## Abstract

Functional depletion of the U1 small nuclear ribonucleoprotein (snRNP) with a 25 nt U1 AMO (antisense morpholino oligonucleotide) may lead to intronic premature cleavage and polyadenylation of thousands of genes, a phenomenon known as U1 snRNP telescripting; however, the underlying mechanism remains elusive. In this study, we demonstrated that U1 AMO could disrupt U1 snRNP structure both *in vitro* and *in vivo*, thereby affecting the U1 snRNP–RNAP polymerase II interaction. By performing chromatin immunoprecipitation sequencing for phosphorylation of Ser2 and Ser5 of the C-terminal domain of RPB1, the largest subunit of RNAP polymerase II, we showed that transcription elongation was disturbed upon U1 AMO treatment, with a particular high phosphorylation of Ser2 signal at intronic cryptic polyadenylation sites (PASs). In addition, we showed that core 3′processing factors CPSF/CstF are involved in the processing of intronic cryptic PAS. Their recruitment accumulated toward cryptic PASs upon U1 AMO treatment, as indicated by chromatin immunoprecipitation sequencing and individual-nucleotide resolution CrossLinking and ImmunoPrecipitation sequencing analysis. Conclusively, our data suggest that disruption of U1 snRNP structure mediated by U1 AMO provides a key for understanding the U1 telescripting mechanism.

Full-length transcription of thousands of genes requires U1 small nuclear ribonucleoprotein (snRNP) to inhibit mRNA 3′ processing at cryptic intronic polyadenylation sites (PASs) of RNA polymerase II (RNAPII) transcripts, which has been termed as “U1 snRNP telescripting” (1, 2, 3, 4). The discovery of U1 snRNP telescripting relies on the usage of a 25 nt antisense morpholino oligonucleotide (AMO) targeting U1 snRNA, whereas the effect of this AMO on the *in vitro* U1 snRNP structure remains unexplored. For *in vivo* mechanism, currently prevalent “U1-CPAF” model states that U1 snRNP transiently associates with CPAF (cleavage and polyadenylation-associated factor), and the U1 AMO targets the 5′ end of U1 snRNA, leading to activation of CPAF near cryptic PAS while maintaining the integrity of U1 snRNP structure (4, 5, 6, 7, 8). This activation is partially achieved by remodeling the CFIm component within CPAF complex (4, 5, 6, 7, 8). Notably, most conclusions are based on quantitative mass spectrometry (MS) analysis. In addition, although previous RNAPII chromatin immunoprecipitation (ChIP) sequencing analyses have suggested that U1 snRNP telescripting is correlated with transcription (3, 5), the underlying mechanisms remain elusive.

Here, we investigated the effect of this U1 AMO on U1 snRNP and unexpectedly found that it could disrupt U1 snRNP structure both *in vitro* and *in vivo*. We further provided evidence that U1 AMO may directly impact the association of U1 snRNP and RNAPII, thereby impacting the transcription activity of the latter. This U1 AMO–induced premature transcription termination might cause premature cleavage and polyadenylation (PCPA). Overall, our data provide an up-to-date understanding of U1 snRNP telescripting mechanism.

## Results

### U1 AMO may affect U1 snRNP structural integrity *in vitro*

**Figure 1:**
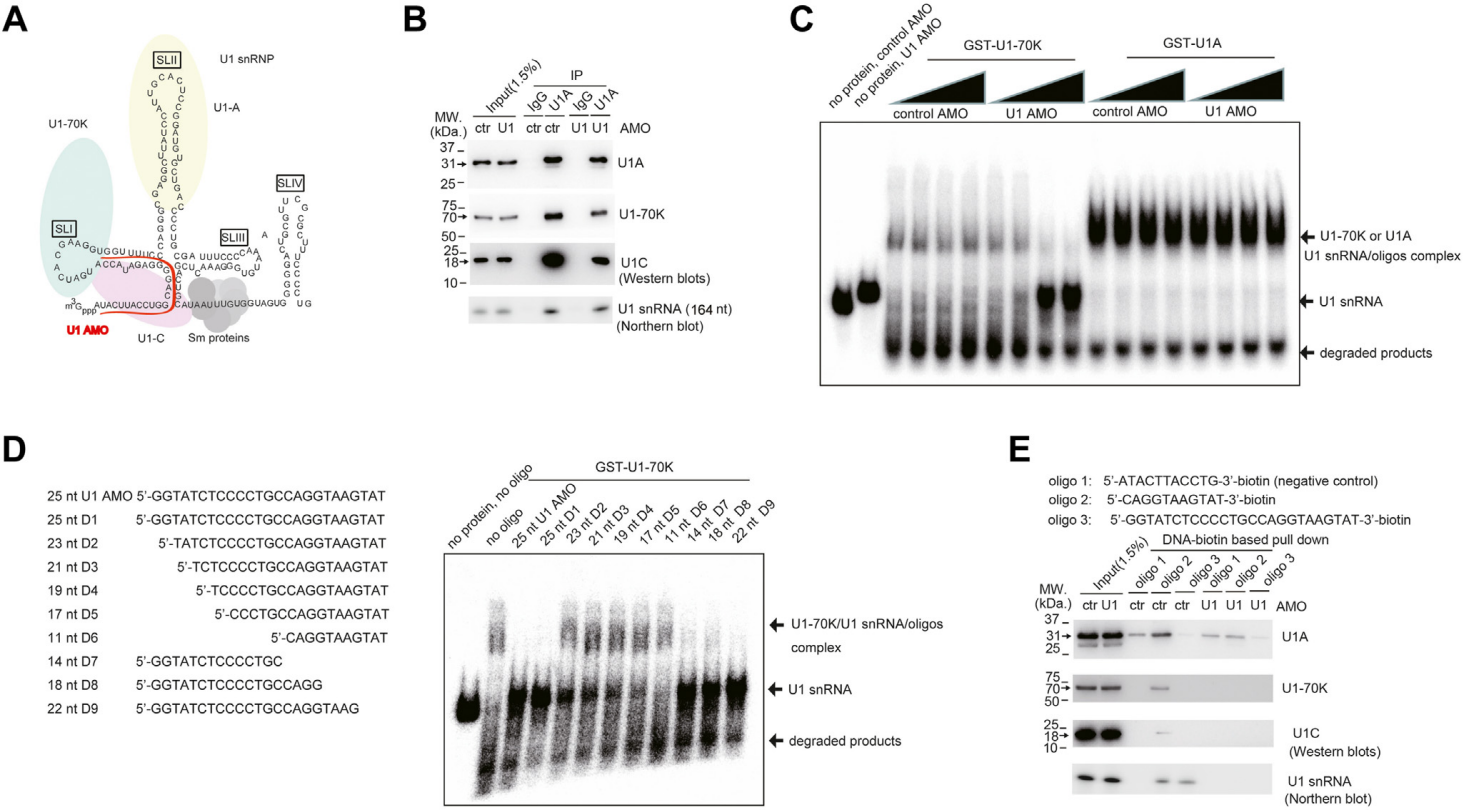
**Effect of U1 AMO on U1 snRNP structure *in vitro*.***A*, schematic representation of components of U1 snRNP complex. *B*, coimmunoprecipitation (IP) analysis using antibodies against control immunoglobulin G (IgG) or U1A in the presence of control or U1 AMO followed by Western blotting analysis (for detection of U1-70K, U1A, and U1C) and Northern blotting analysis (for detection of U1 snRNA). *C*, gel mobility shift assays using detectable amount of radiolabeled U1 snRNA (about 0.2 μM) and recombinant GST-U1-70K (5 μM) or GST-U1A (5 μM) protein in the presence of control or U1 AMO (0, 0.02, 0.2, and 2 μM). The protein–RNA complex, free RNA, and degraded RNA products are indicated with *arrows*. *D*, gel mobility shift assays using detectable amount of radiolabeled U1 snRNA (about 0.2 μM) and recombinant GST-U1-70K protein (5 μM) in the presence of U1 AMO or DNA oligos (2 μM). The protein–RNA complex, free RNA, and degraded RNA products are indicated with *arrows*. The sequences of the DNA oligonucleotides are listed in the *left panel*. *E*, DNA–biotin-based pull-down assays using the indicated oligos and HeLa NE followed by Western blotting analysis (for detection of U1-70K, U1A, and U1C) and Northern blotting analysis (for detection of U1 snRNA). The sequence of the biotin-labeled DNA oligonucleotides is listed in the *upper panel*. AMO, antisense morpholino oligonucleotide; GST, glutathione-*S*-transferase; NE, nuclear extract; snRNP, small nuclear ribonucleoprotein.

We hypothesized that the 25 nt U1 AMO, routinely used in previous experiments for functional knockdown of U1 snRNA, might disrupt U1 snRNP structural integrity based on two observations. First, in the nuclear extract (NE) prepared from HeLa cells, coimmunoprecipitation (co-IP) analysis using anti-U1C antibody showed that U1-70K and U1A, two other U1 snRNP–specific proteins, were significantly downregulated in the IPed sample using U1 AMO–treated NE (9). Second, it is well established that U1-70K can bind U1 snRNA stem loop 1 (SL1) (Fig. 1*A*) (10, 11, 12), which is close to the region where 25 nt U1 AMO binds, and U1C was also reported to be associated with U1 snRNA at the 5′ end within U1 snRNP complex (13). Therefore, U1 AMO might interfere with the associations of U1 snRNA and U1-70K–U1C.

**Supporting Figure S1:**
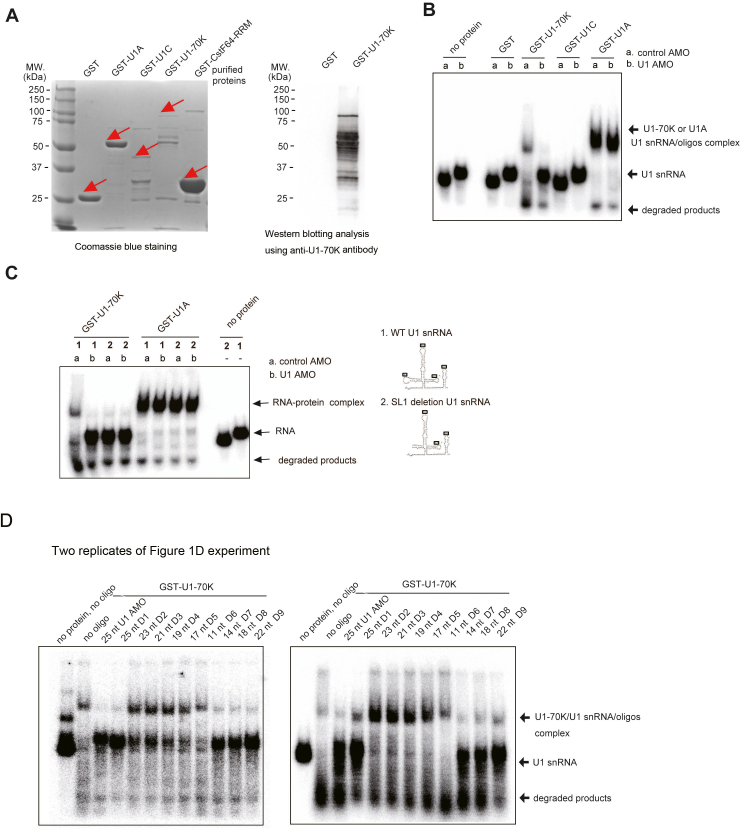
*A*, commassie blue staining of purified recombinant GST-U1-70K, GST-U1A, GST-U1C and GST-CstF64-RRM (RNA recognition motif) proteins. Red arrow in each lane indicates the band of the expressed target protein (Approximately 10μg proteins were loaded for each lane). Western blotting analysis was carried out to confirm that the purified GST-U1-70K protein could be recognized by an anti-U1-70K antibody (Santa cruz; sc-390988). GST protein serves as negative control. *B*, gel mobility shift assays using detectable amount of radiolabeled U1 snRNA (about 0.2 μM) and GST protein or recombinant GST-U1-70K (5 μM), GST-U1C (5 μM) protein or GST-U1A (5 μM) in the presence of control or U1 AMO (2 μM). ‘a’ denotes control AMO and ‘b’ denotes U1 AMO. *C*, gel mobility shift assays using purified GST-U1-70K/GST-U1A and wild type U1 snRNA/SL-1 deletion mutant, in the presence of control/U1 AMO. ‘a’ denotes control AMO and ‘b’ denotes U1 AMO. ‘1’ represents wild type U1 snRNA and ‘2’ represents stem loop1 (SL1) deletion U1 snRNA mutant. The deleted SL1 sequence is ‘-UGAUCACGAAGG-’. The protein-RNA complex, free RNA, and degraded RNA products are indicated with arrows. Protein/RNA/AMO concentration is the same as (*B*). *D*, two replicates of Figure 1*D*. Please see the Figure 1*D* legend for details.

We first performed a similar co-IP experiment to test the aforementioned hypothesis. Instead of an anti-U1C antibody, we used an anti-U1A antibody. Consistent with earlier findings (9), we observed a mild decrease in U1-70K protein and an apparent reduction in U1-C protein in the IPed products for U1 AMO–treated samples (Fig. 1*B*). The results suggest that U1 AMO might interfere with protein–protein interactions within the U1 snRNP complex in the NE. To test if U1 AMO could affect protein–RNA interactions within the U1 snRNP complex, we took advantage of the knowledge that U1-70K and U1A could directly bind U1 snRNA and performed a gel mobility shift assay in the presence of control and U1 AMO. Recombinant glutathione-*S*-transferase (GST)-U1-70K/U1A/U1C proteins were prepared using the constructs (gift from Dr Marc-Étienne Huot, Université Laval; confirmed by Sanger sequencing) characterized in previous work (14). As expected, recombinant GST-U1-70K and U1A proteins formed stable protein–RNA complexes with U1 snRNA in the presence of control AMO (Fig. S1, *A* and *B*). However, we found that U1 AMO could efficiently block U1-70K binding of U1 snRNA even at low concentration (0.2 μM), wherein U1-70K–U1 snRNA was near a 1:1 ratio (Fig. 1*C*). To further substantiate that the supershifted band corresponds to U1-70K–U1 snRNA complex, the SL1 deletion mutant U1 snRNA was prepared and subsequently used for the same gel mobility shift assay. Indeed, a sharp decrease in the signal of the supershifted band was observed (Fig. S1*C*). In contrast, the protein–RNA interaction of GST-U1A and U1 snRNA was unaffected in the tested conditions.

We predicted that U1 AMO might block U1-70K binding through steric hindrance. We employed a DNA oligo harboring the same sequence with 25 nt U1 AMO and performed the aforementioned gel shift assay to test this. Indeed, similar to AMO, 25 nt antisense DNA oligo could also efficiently block U1-70K–U1 snRNA association (Fig. 1*D*). The results motivated us to subsequently test the blocking efficiency of a series of truncated forms of U1 antisense DNA. As expected, the shortening of the 5′ end of antisense DNA gradually alleviated the inhibitory effect, which coincided with the fact that the binding region of the 5′ end of antisense DNA was close to U1 snRNA SL1; however, the 3′ end was unaffected (Figs 1*D* and S1*D*).

To consolidate the aforementioned steric hindrance model, we applied a previously reported biotin-based antisense oligo pull-down method to enrich the U1 snRNP complex from HeLa NE (15). The 11 nt biotin-labeled DNA, which is complementary to the free 5′ end of U1 snRNA, could enrich significant amount of U1-70K and U1C in the pull-down assay (Fig. 1*E*). In contrast, the 25 nt biotin-labeled DNA pull-down sample returned undetectable U1-70K and U1C proteins. Northern blotting analysis of U1 snRNA demonstrated that both biotin–DNA oligos could enrich U1 snRNA with similar efficiency (Fig. 1*E*), excluding the possibility that the observed difference resulted from differential U1 snRNA pull-down efficiency (Fig. 1*E*).

### U1 AMO may affect U1 snRNP structural integrity *in vivo* and impact RNAPII activity

**Figure 2:**
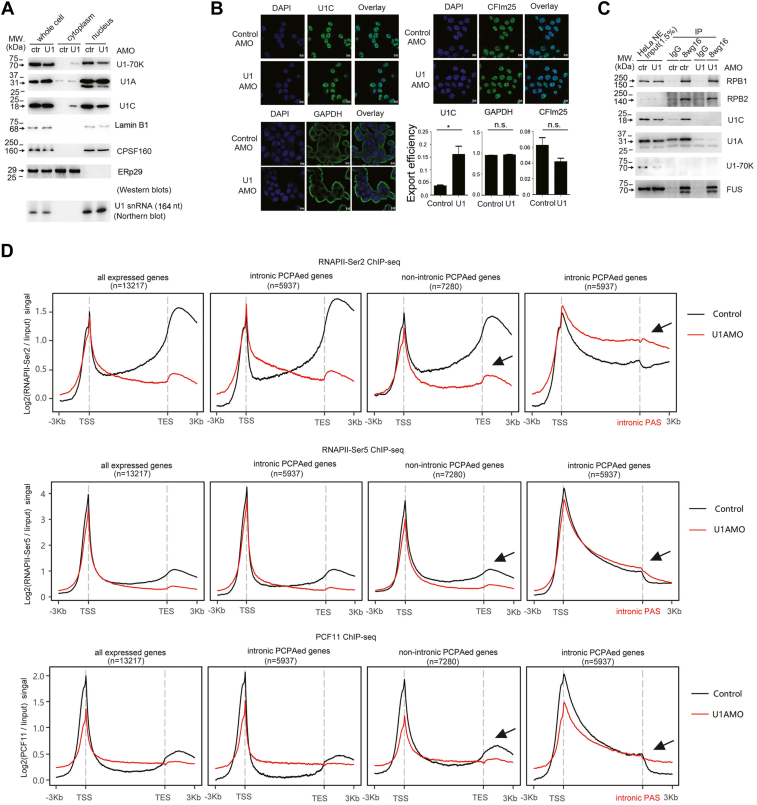
**Effect of U1 AMO on U1 snRNP structure *in*** ***vivo*.** All the experiments were performed using HeLa cells. *A*, Western blotting analysis and Northern blotting analysis (for detection of U1 snRNA) to examine the subcellular distribution of U1 snRNP components and indicated protein markers upon control or U1 AMO transfection. Ctr represents control AMO–treated cells, and U1 represents U1 AMO–treated cells. *B*, immunostaining of U1C, GAPDH, and CFIm25 proteins in control and U1 AMO–treated cells (Ctr represents control AMO, and U1 represents U1 AMO). The scale bar of 10 μm is shown in the *right bottom* of the picture. Graphs show the percentages of exported efficiencies (cytoplasm/total immunostaining signal) quantified using ImageJ software. The results are from 20 different randomly selected cells from three independent microscope images. Data are shown as mean ± SEM. Student's *t* test was performed to examine the significance of the difference. ∗*p* < 0.05; ns, nonsignificant change. *C*, coimmunoprecipitation (IP) analysis using control immunoglobulin G (IgG) or 8wg16 antibodies in the presence of control or U1 AMO followed by Western blotting analysis to detect the indicated proteins. The input is 1.5% of the total lysates. *D*, metagene plots of RNAPII Ser2P/Ser5P and PCF11 chromatin immunoprecipitation (ChIP-Seq) reads in control and U1 AMO–treated HeLa cells for all actively expressed genes (n = 13,217), intronic PCPAed genes (n = 5937), and other nonintronic PCPAed genes (n = 7280). For intronic PCPAed genes, a second metagene plot was made for each ChIP-Seq by replacing the default TES (transcription end site) with intronic PCPA sites. The *arrows* are used to emphasize the difference of the ChIP-Seq profile near TES between the two groups of genes. Control represents control AMO–treated cells, and U1 represents U1 AMO–treated cells. PCPA, premature cleavage and polyadenylation; RNAPII, RNA polymerase II.

**Supporting Figure S2:**
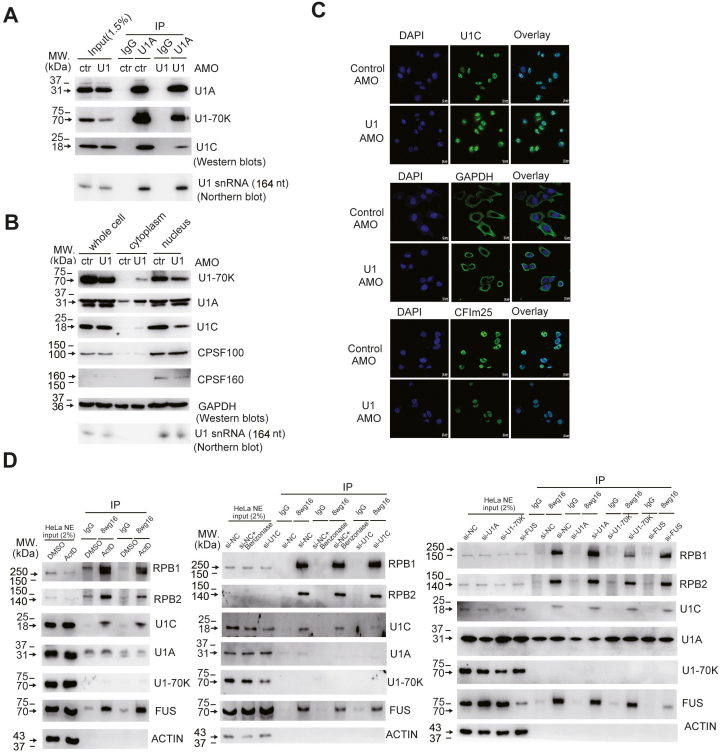
*A*, co-immunoprecipitation (IP) analysis were performed using NE prepared from HeLa cells transfected with control or U1 AMO, and antibodies against control IgG or U1A. Western blotting analysis (for detection of U1-70K, U1A and U1C) and Northern blotting analysis (for detection of U1 snRNA) were subsequently carried out to determine the relative Co-IP efficiency. The input is 1.5% of HeLa NE used for IP. *B*, Western blotting analysis (for detection of U1-70K, U1A and U1C) and Northern blotting analysis (for detection of U1 snRNA) to examine the subcellular distribution of U1 snRNP components upon control or U1 AMO transfection in SW480 cells. The indicated proteins (CPSF160, CPSF100 and GAPDH) serve as controls. *C*, immunostaining of U1C, GAPDH and CFIm25 proteins in control and U1 AMO treated SW480 cells. The experiments have been carried out three times, and a representative picture is shown. 10 μm scale bar is shown in the right bottom of the picture. *D*, co-immunoprecipitation (IP) analysis using control IgG and 8wg16 antibodies under various conditions (Actinomycin D treatment; Benzonase treatment; siRNAs transfection targeting negative control/U1A/U1C/U1-70K/FUS protein; ActD:Actinomycin; NC: negative control) followed by Western blotting analysis (for detection of indicated proteins). The input is 2% of HeLa NE used for IP. The experiments have been carried out three times, and a representative picture is shown.

To investigate if U1 AMO could affect U1 snRNP structure *in vivo*, we performed control and U1 AMO transfection in HeLa cells and subsequently prepared NE for the same aformentioned co-IP analysis. It appeared that the U1 inhibitory effect is more pronounced than the results obtained in the aforementioned experiment (Figs. 1*B* and S2*A*). This was partly because the nucleus contains less U1-70K and U1C protein upon U1 AMO transfection. To confirm this, we examined their expression in the whole cells, nucleus, and cytoplasm, respectively. The results demonstrated that U1 AMO treatment consistently led to an apparent accumulation of U1-70K–U1C proteins in the cytoplasm and a corresponding downregulation of U1-70K–U1C in the nucleus (Fig. 2*A*). In contrast, their overall protein expression was not much disturbed. It must be noted that the observed difference was not caused by potential crosscontamination between cytoplasm and nucleus extracts, as demonstrated by the expression pattern of several nuclear and cytoplasmic protein markers, including lamin B1 and Erp29. In addition, U1 AMO did not appear to cause the U1 snRNA accumulation in the cytoplasm, as revealed by the Northern blotting analysis (Fig. 2*A*).

To complement the results observed in the Western blotting analysis, we performed immunostaining for U1C. An apparent immunostaining signal was observed in the cytoplasm for U1 AMO–treated cells, whereas it was exclusively localized in the nucleus for control cells (Fig. 2*B*). Further quantification of U1C export efficiency by calculating the ratio of intensity signal in the cytoplasm and whole cells confirmed the significance of the change (Fig. 2*B*). These results suggested that U1 AMO transfection significantly affected the overall U1 snRNP structural integrity *in vivo*. We also carried out the aforementioned experiments in another human cell line SW480, and the results were the same obtained in HeLa cells (Fig. S2, *B* and *C*). Overall, we showed that U1 AMO could disrupt U1 snRNP structure *in vivo*, arguing against the prevalent U1-CPAF model that U1 snRNP structure is not perturbed upon U1 AMO transfection (6).

Recent studies have shown that U1 snRNP associates with RNAPII (9, 16, 17, 18); we next investigated if this association is disrupted in U1 AMO–treated cells. Using a well-established antibody (8wg16) targeting the CTD (C-terminal domain) of RPB1, the largest subunit of RNAPII, our co-IP analysis confirmed that RNAPII may interact with U1C in the control HeLa cells (Fig. 2*C*). This interaction might be independent of nucleic acids, gene transcription, and FUS protein, as shown by the similar results under the treatment of benzonase, actinomycin D, or siRNAs targeting FUS protein (Fig. S2*D*). As expected, this interaction was significantly disrupted in U1 AMO–treated HeLa cells (Fig. 2*C*).

**Supporting Figure S3:**
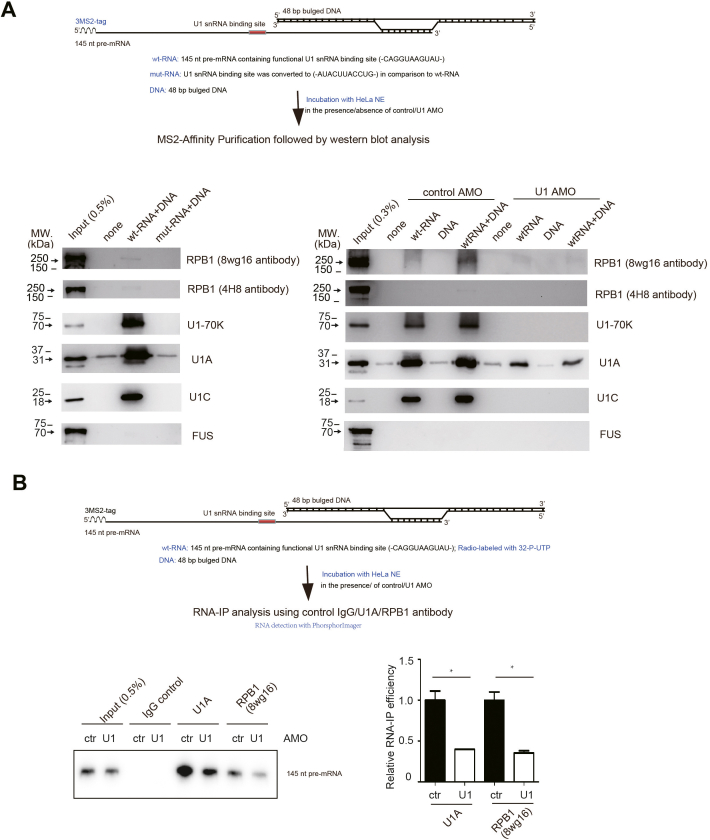
*A*, MS2-tagged RNA-based pull-down assay using HeLa NE in the presence of control or U1 AMOs followed by Western blotting analysis to examine the abundance of indicated proteins in the pull-down sample. ‘wt RNA’ indicates the 145 nt wild type pre-mRNA containing canonical U1 snRNA binding site; ‘mut RNA’ indicates the 145 nt mutant pre-mRNA containing no functional U1 snRNA binding site; ‘DNA’ indicates the 48 bp bulged DNA double strand; ‘DNA+RNA’ indicates that both pre-mRNA and DNA were added in the pull-down assay; ‘none’ indicates that no nucleic acids were added; all the wt, mutant pre-RNA and DNA sequences were listed in Table S4. The working concentration of pre-mRNA and NE used in the pull-down assay are exactly the same as Figure 3*A*. DNA concentration is the same as pre-mRNA. AMO concentration is 10 μM. *B*, radio-labeled wt pre-mRNA and DNA (cold) was incubated with HeLa NE in the presence of control or U1 AMO. The working concentration of pre-mRNA/DNA and NE is the same as Figure 3*A*. IgG, U1A or RPB1 antibodies were used to capture their associated pre-mRNA in the mixture. The RNA IP efficiencies were quantified from three independent experiments by calculating the ratio of IPed signal of radio-labeled pre-mRNA relative to the input signal. Data are shown as mean ± SEM. Student's t-test was performed to examine the significance of the difference. ∗*p* < 0.05. Ctr represents control AMO and U1 represents U1 AMO.

We further asked if U1 AMO has a direct impact on RNAPII transcription activity. To examine this, we first utilized an *in vitro* reconstitution system previously applied to characterize the structure of U1-snRNP–RNAPII (19). In this system, a synthesized pre-mRNA was hybridized to bulged DNA template, mimicking the gene transcription process (Fig. S2*A*). Instead of using purified U1 snRNP and RNAPII components, we used HeLa NE for *in vitro* assay. Indeed, by adopting the well-established MS2-tagged RNA affinity purification method (19, 20), U1 snRNP and RPB1 could be detected in the pull-down sample (Fig. S3*A*). Mutation of U1 snRNA binding site apparently decreased the binding of both U1 snRNP and RPB1, indicating that U1 snRNP contributes to the association of RPB1 with DNA–pre-mRNA. As expected, little U1 snRNP proteins were detected in the presence of U1 AMO. Significantly, decreased pull-down efficiency was observed for RPB1 when U1 AMO was added in the NE compared with the control AMO, as evidenced by Western blotting analysis (Fig. S3*A*). These results indicated that RPB1 tended to fall off DNA–pre-mRNA hybrid in the presence of U1 AMO. To further substantiate this point, pre-mRNA was radiolabeled in the aforementioned reaction mixture (containing both DNA and wildtype pre-mRNA), and RNA-IP analysis was carried out using RPB1 antibody. Similar as U1A, RPB1 showed a significantly weaker association with pre-mRNA upon U1 AMO treatment (Fig. S3*B*).

**Supporting Figure S4:**
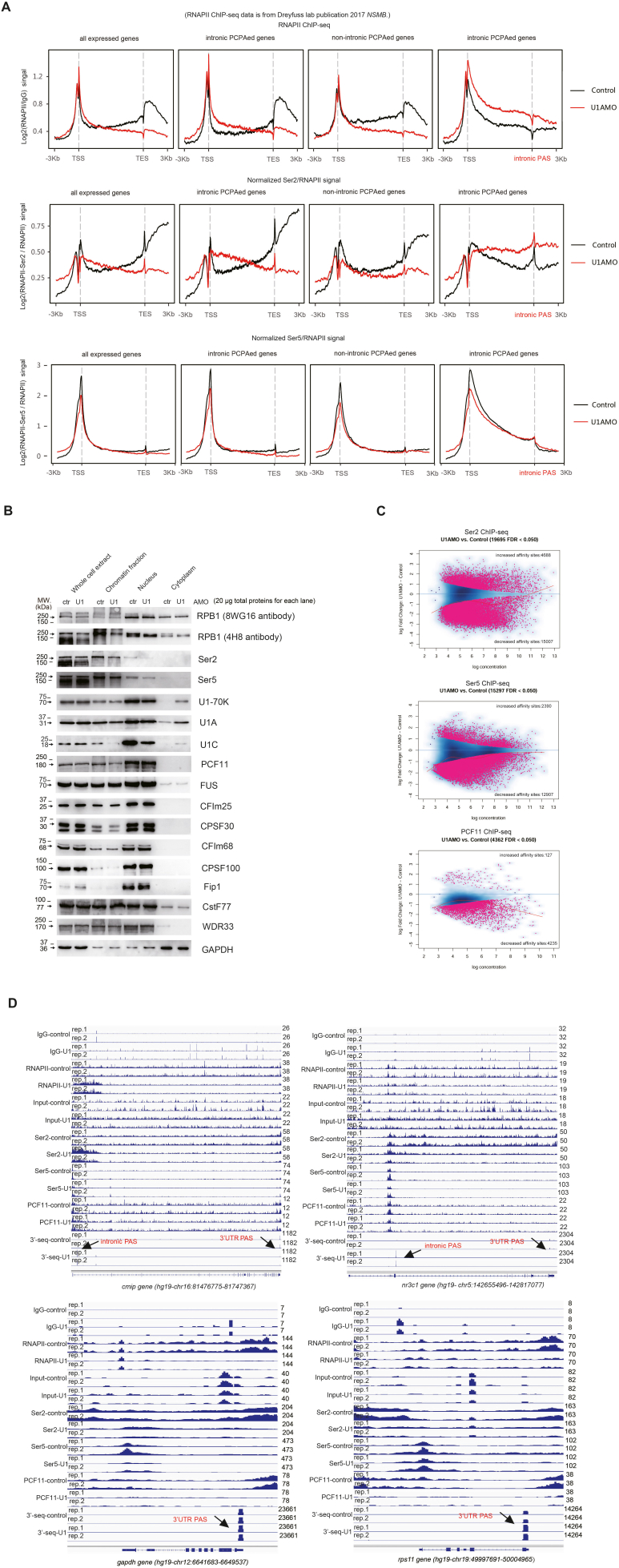
*A*, meta-gene plots of RNAPII and normalized Ser5P/Ser2P (normalized to RNAPII) ChIP-seq reads in control and U1 AMO treated HeLa cells for all actively expressed genes (n = 13,217), intronic PCPAed genes (n = 5937), and other non-intronic PCPAed genes (n = 7280). For intronic PCPAed genes, a second meta-gene plot was made for each ChIP-seq by replacing the default TES (transcription end sites) with intronic PCPA site. RNAPII ChIP-seq data were downloaded from Dreyfuss publication (2017, *NSMB*). Control represents control AMO and U1 represents U1 AMO. *B*, Western blotting analysis of indicated proteins in corresponding subcellular fractions (whole cell extracts, chromatin, nucleus and cytoplasm) prepared from HeLa cells treated with control or U1 AMO. Approximately 20 μg total proteins were loaded for each lane. *C*, MA-plots showing the significantly differential binding sites in the presence of U1 AMO based on the original ChIP-seq data. Peak calling was performed using MACS3 software and DiffBind package was used to identify the differential binding events. The number of decreased affinity sites (dots below the zero line; colored red) and increased affinity sites (dots above the zero line; colored red) are shown in the plot. Dots colored blue indicates the binding sites showing a statistically non-significant change between control and U1 AMO treated sample. *D*, IGV track screen shots showing ChIP-seq and 3′-seq results for PCPAed genes (*cmip*, *nr3c1*) and non-PCPAed genes (*gapdh, rps11*) in control and U1 AMO treated HeLa cells. Rep.1 and rep.2 represent results from two individual biological replicate experiment. The intronic PAS and/or 3′-UTR PAS for specific gene has been indicated with arrow.

We subsequently investigated the impact of U1 AMO on two RNAPII CTD modifications, namely Ser5P and Ser2P, by ChIP-Seq analysis (two biological replicates for each sample), given their direct links with active gene transcription *in vivo* (21, 22, 23). Consistent with the previous RNAPII ChIP-Seq analysis (3), upon U1 AMO treatment, a progressive decrease in RNAPII Ser5P and Ser2P signal was observed for all the actively expressed genes (Figs. 2*D* and S4*A*; Table S1). This result is in line with the observation that detectable decreases were observed for Ser5P and Ser2P in chromatin fraction upon U1 AMO transfection (Fig. S4*B*). To gain insight into U1 snRNP telescripting, we divided the actively expressed genes into intronic PCPAed genes and nonintronic PCPAed genes, using our recently published 3′-seq data and QuantifyPoly(A) pipeline (24, 25). In total, we found that the usage of 21,248 intronic PASs distributed among 5937 genes was significantly elevated upon U1 AMO treatment (Log2 [fold change] >1; *p* < 0.05) (Table S2). Therefore, intronic PCPAed genes hereafter refer to these 5937 genes, and nonintronic PCPAed genes refer to other 7280 genes (in total 13,217 genes), if not indicated otherwise. Indeed, an apparent difference between the two subgroups was observed (Fig. 2*D*). For intronic PCPAed genes, a sharp increase in RNAPII Ser2P signal, together with an apparent increase in RNAPII signal (Fig. S4*A*), was observed between the TSS and intronic PAS, implicating a scenario that RNAPII movement tended to pause toward intronic PAS, thereby providing a time window for intronic PAS processing. For those non-PCPAed genes, RNAPII directly fell off transcribing region in U1 AMO–treated cells (Figs. 2*D* and S4*A*). In addition, we performed ChIP-Seq analysis for PCF11, a transcription termination–associated factor (26, 27), and a similar discrepancy was observed for the two groups of genes (Figs. 2*D* and S4*C*). Fig. S4*D* shows each group's ChIP-Seq(s) profiles for several representative genes.

### Core 3′ processing factors CPSF/CstF play roles in intronic PAS processing

**Figure 3:**
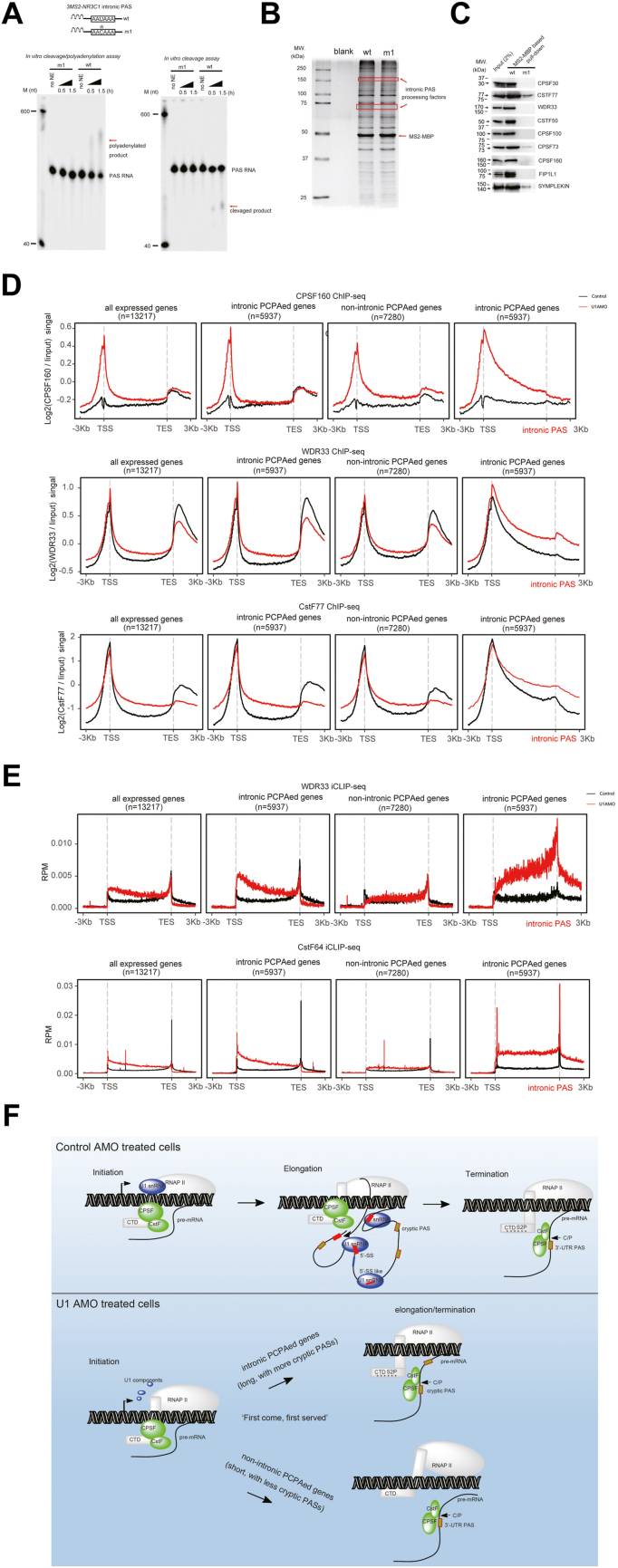
**Effect of U1 AMO on the recruitment of core intronic PAS processing factors onto chromatin and pre-mRNA.***A*, *in vitro* cleavage/polyadenylation and *in vitro* cleavage assays using HeLa NE and RNA substrates derived from intronic PAS of *nr3c1* gene. Three repeats of MS2 sequences were added to the 5′ end of the PAS RNA to facilitate the process of protein purification. As negative control, a mutant PAS RNA harboring a point mutation in the core hexamer AAUAAA was prepared simultaneously. The PAS RNA sequences are listed in Table S4. The polyadenylated or cleaved RNA products are indicated with *red arrows*. *B*, silver staining of protein complexes purified from HeLa NE using the indicated PAS RNAs. Potential intronic PAS processing factors, which are indicated with *red arrows*, could be purified by wt but not m1 PAS RNAs. *C*, validation of mass spectrometric results by Western blotting analysis using antibodies against indicated proteins. The input is 2% of the total lysates. *D*, metagene plots of CPSF160, WDR33, and CSTF77 chromatin immunoprecipitation (ChIP-Seq) reads in control and U1 AMO–treated HeLa cells for all actively expressed genes (n = 13,217), intronic PCPAed genes (n = 5937), and other nonintronic PCPAed genes (n = 7280). Control represents control AMO–treated HeLa cells, and U1 represents U1 AMO–treated HeLa cells. *E*, metagene plots of WDR33 and CSTF64 iCLIP-Seq reads in control and U1 AMO–treated HeLa cells for all actively expressed genes (n = 13,217), intronic PCPAed genes (n = 5937), and other nonintronic PCPAed genes (n = 7280). For intronic PCPAed genes, a second metagene plot was made for each ChIP-Seq/iCLIP-Seq by replacing the default TES (transcription end site) with intronic PCPA sites. Control represents control AMO–treated HeLa cells, and U1 represents U1 AMO–treated HeLa cells. *F*, a schematic diagram summarizing the key finding in our study. The details are described in the main body of the text. iCLIP-Seq, individual-nucleotide resolution CrossLinking and ImmunoPrecipitation sequencing; PCPA, premature cleavage and polyadenylation.

**Supporting Figure S5:**
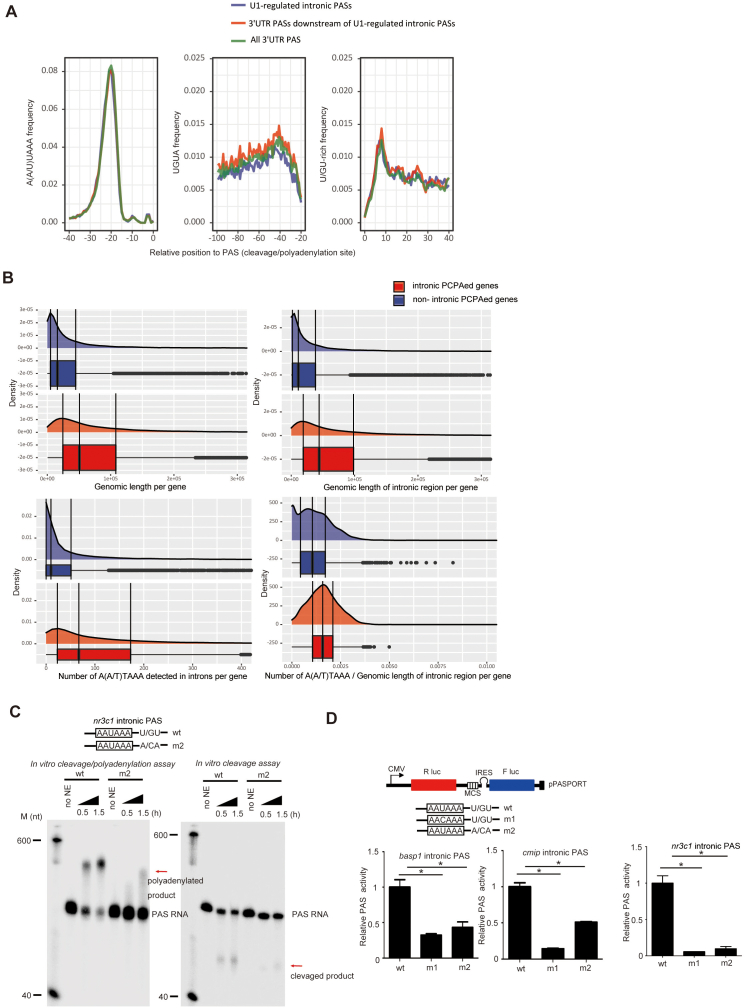
*A*, density plot showing the frequencies of 'A(A/U)UAAA', 'U/GU' and 'UGUA' elements near the indicated PAS groups. *B*, boxplots showing the distribution of gene size/intronic size/number of A(A/U)UAAA/normalized number of A(A/U)UAAA of intronic PCPAed genes (n = 5937) and non-intronic PCPAed genes (n = 7280). Boxplot midlines represent the median, box limits span the first and third quartiles. Two-samples Wilcoxon rank sum test was used to examine the significance of the difference. ∗*p* < 2.2e−16. *C*, *in vitro* cleavage/polyadenylation and *in vitro* cleavage assays using HeLa NE and RNA substrates derived from intronic PAS of *nr3c1* gene. To create CstF64 binding mutant, downstream U/GU rich sequences were replaced with A/CA sequences. The sequences of wild type PAS RNA (wt) and CstF64 binding mutant PAS RNA (m2) are listed in Table S4. The polyadenylated or cleaved RNA products are indicated with red arrows. The experiments have been carried out twice, and a representative picture is shown. The left panel is the result of *in vitro* cleavage/polyadenylation assay, and the right panel is the result of *in vitro* cleavage assay. *D*, measurement of the processing efficiencies of *basp1*, *cmip*, *nr3c1* intronic PAS RNAs and two mutants using pPASPORT system (please see ref. (30) for the details of this system). wt represents wild type PAS RNA; m1 represents CPSF binding mutant; m2 represents CstF64 binding mutant. Experiments were performed three times and the standard deviations are shown in error bars. Student's *t*-test was performed to examine the significance of the difference. ∗*p* < 0.05.

Furthermore, we wished to identify 3′ processing machinery associated with the processing of intronic PASs. Although it is predicted that intronic PASs and canonical 3′ UTR PASs share common 3′ processing machinery based on common PAS RNA cis-elements (Fig. S5, *A* and *B*) (24, 28), more direct experimental evidence still needs to be provided. To this goal, we adopted a similar RNA affinity approach previously used to purify the canonical PAS processing complex (20, 29). Briefly, three copies of MS2 hairpin were fused to the intronic PAS of the *nr3c1* gene (Fig. 3*A*), which is a *bona fide* PCPA target upon U1 AMO treatment (Fig. S4*D*). To make a negative-control RNA substrate, the core hexamer AAUAAA within PAS was mutated to AACAAA. The RNA substrate was incubated with HeLa NE, and the mRNA 3′ processing complex was purified using an MS2 tag. After RNA pull-down, silver staining and quantitative protein analysis by MS were performed to estimate the abundance of 3′ processing factors. Indeed, MS analysis followed by Western blotting validation indicated that most of the top hits were known CPSF/CstF factors (Fig. 3, *B* and *C*; Table S3). By mutating the CPSF/CstF binding sites of several intronic PASs, we further confirmed that CPSF/CstF play positive roles in processing of the intronic PAS using *in vitro* mRNA 3′’ processing assays and a luciferase reporter assay that was reported previously (Fig. S5, *C* and *D*) (30). Our results therefore provided more direct experimental evidence that known core 3′ processing factors CPSF/CstF were involved in intronic PAS processing upon U1 AMO treatment.

### U1 AMO treatment manipulates CPSF/CstF cotranscriptionally

**Supporting Figure S6:**
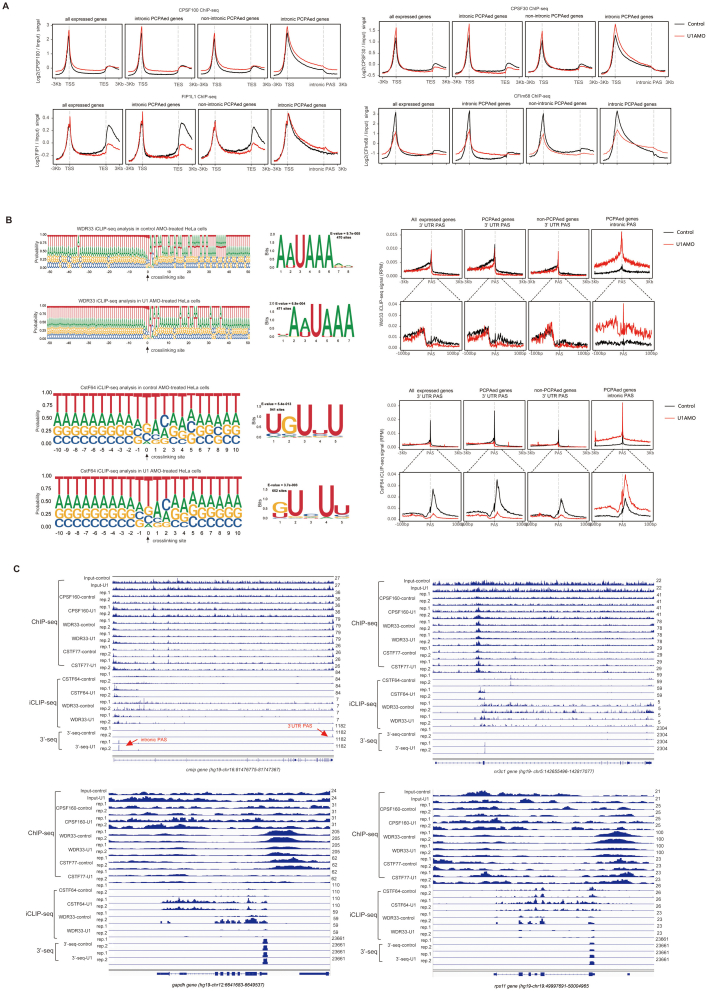
*A*, meta-gene plots of CPSF100, CPSF30, FIP1L1, and CFIm68 ChIP-seq reads in control and U1 AMO treated HeLa cells for all actively expressed genes (n = 13,217), intronic PCPAed genes (n = 5937), and other non-intronic PCPAed genes (n = 7280). For intronic PCPAed genes, a second meta-gene plot was made for each ChIP-seq by replacing the default TES (transcription end sites) with intronic PCPA sites. If multiple PCPA sites were detected for a given gene, the one showing the most significant PCPA was chosen to create the plot. Control represents control AMO and U1 represents U1 AMO treated sample. *B*, sequence logo based on all reproducible crosslinking nucleotides and 50 nt (WDR33)/10 nt (CSTF64) on each side . MEME (Multiple EM for Motif Elicitation) of the top 1000 WDR33/CSTF64 binding sites (Left panel). The most enriched motifs from each group are shown on the middle panel. The right panel shows the iCLIP-seq profile near the indicated PAS regions (all gene 3′-UTR PAS; intronic PCPAed genes 3′UTR PAS; non-intronic PCPAed gene 3′ UTR PAS; intronic PASs of intronic PCPAed genes). Control represents control AMO and U1 represents U1 AMO treated sample. *C*, IGV track screen shots showing CPSF160/WDR33/CSTF77 ChIP-seq(s), WDR33/CSTF64 iCLIP-seq(s) and 3′-seq results for PCPAed genes (*cmip* and *nr3c1*) and non-PCPAed genes (*gapdh* and *rps11*) in control and U1 AMO treated HeLa cells. Rep.1 and rep.2 represent results from two individual biological replicate experiment. In the picture on the right, the numbers indicate maximum value of the reads used to display track data.

We hypothesized that CPSF/CstF associations with transcribed genes might be manipulated under the U1 AMO treatment condition. ChIP-Seq(s) were performed to test most of known core CPSF/CstF factors (two biological replicates for each sample). Consistent with the current cotranscriptional recruitment model of 3′ processing factors (31, 32), a sharp peak at TSS in the ChIP-SSeq profile for WDR33, CPSF30, CPSF100, FIP1, and CstF77 in control cells was detected. In contrast, another major peak was observed near transcription end site only for WDR33, FIP1, and CstF77 (Figs. 3*D* and S6*A*), coinciding with their roles in canonical PAS processing. Overall, we found that most of the ChIP-Seq profiles investigated for CPSF/CstF factors showed detectable changes in U1 AMO–treated cells (Figs. 3*D* and S6*A*). Among them, CPSF160 showed the most striking change, and it appeared that U1 AMO activated CPSF160 association with genomic regions of both intronic PCPAed and remaining actively transcribed genes. Wdr33, CPSF100, and CstF77 also showed mildly elevated signals across the gene body for all the actively expressed genes. However, for CPSF30 and FIP1, a slight increase in binding signal was only observed for intronic PCPAed genes, whereas the opposite trend was observed for non-PCPAed genes.

We subsequently mapped protein–RNA interactions *in vivo* by individual-nucleotide resolution CrossLinking and ImmunoPrecipitation sequencing (iCLIP-Seq) for two canonical CPSF/CstF factors, WDR33 and CstF64, before and after U1 AMO treatment (two biological replicates for each sample). Our iCLIP-Seq(s) were reliable based on the specific binding patterns and enriched motifs (Figs. 3*E* and S6*B*) (30, 33, 34). Two major observations were made from the iCLIP-Seq analysis between the control and U1 AMO–treated cells. First, for intronic PCPAed genes, an elevated iCLIP-Seq signal was detected across the gene body for both WDR33 and CstF64 upon U1 AMO treatment (Fig. 3*E*), consistent with the results obtained from ChIP-Seq data and suggested the U1 AMO enhanced CPSF/CstF associations with DNA–pre-mRNA cotranscriptionally. Second, for non-PCPAed genes, the change in iCLIP-Seq signal value was not as apparent as that in PCPAed genes (Fig. 3*E*), consistent with the results from bioinformatics analysis that non-PCPAed transcripts tend to be short and have less PAS motifs recognized by CPSF/CstF (Fig. S5*B*). Fig. S6*C* shows several representative examples illustrated by snapshots of mapped reads using the IGV software (Broad Institute).

## Discussion

To summarize this study, a schematic model has been proposed in Figure 3*F*. In addition to splicing, U1 snRNP could be a key player in sustaining transcription elongation by associating with RNAPII in control cells. Although CPSF/CstF could be loaded onto chromatin during the transcription initiation step, they are not fully assembled onto pre-mRNA 3′UTR PAS until RNAPII reaches the end of transcription unit, wherein RNAPII CTD Ser2 is heavily phosphorylated. In U1 AMO–treated cells, the integral U1 snRNP complex might fall apart (some of the U1-specific proteins could be exported to the cytoplasm), which affected its association with RNAPII and ultimately affected transcription elongation. Based on the well-accepted “first come, first served” cotranscriptional mRNA 3′ processing model (35, 36), the CPSF/CstF complex could be fully assembled onto cryptic PASs of intronic PCPAed genes once U1 AMO–triggered premature transcription termination occurs. The intronic mRNA 3′ processing, RNAPII pausing, and RNAPII CTD Ser2P could be reciprocally regulated near intronic PAS (37, 38, 39). In contrast, nonintronic PCPAed genes tend to be short and have less intronic PAS, and their 3′ end formation takes place soon after the 3′ UTR PAS is transcribed.

To elucidate the U1 snRNP telescripting mechanism, the previous U1-CPAF activation model has focused on the compositions and functions of U1 snRNP–associated factors (4, 5, 6, 7, 8). Unlike this model, we found that the 25 nt U1 AMO could significantly affect the protein–protein interaction and protein–RNA interaction within the U1 snRNP complex, both *in vitro* and *in vivo*. Furthermore, we provided evidence that the activation of CPAF upon U1 AMO treatment is associated with RNAPII pausing and RNAPII CTD Ser2P. Although CFIm68 may be involved in this process, as suggested by U1-CPAF model (6), RNAPII CTD and Ser2P might also be involved, given that RNAPII CTD and Ser2P play key roles in cotranscriptional mRNA 3′ processing (37, 38, 39). In addition, we showed that the movement of core 3′ processing factors CPSF/CstF across gene body is globally modified upon U1 AMO transfection, providing additional insight into the mechanism of U1 snRNP telescripting.

Our study has also raised several interesting questions for future investigation. First, consistent with current cotranscriptional mRNA 3′ end processing model (32, 37, 38, 39), RNAPII pausing and high RNAPII Ser2P density were observed near the intronic PAS for those intronic PCPAed genes upon U1 AMO treatment, whereas the trend was not apparent for non-PCPAed genes (Fig. 2*D*), indicating that Ser2P and 3′ end processing might be uncoupled during the process of transcription termination for these non-PCPAed genes under U1 AMO condition, which adds a layer of complexity to the regulatory mechanism of cotranscriptional mRNA 3′ end processing. Second, although our study and previous studies have suggested that the U1 snRNP and RNAPII might have physical interactions (Fig. 2*C*) (9, 16, 17, 18); it remains unclear if this association is direct or indirect, particularly in a cellular context, which may enable us to understand how U1 AMO caused the differential RNAPII activity.

Finally, although we have provided data that U1 AMO disrupts U1 snRNP structure and affects transcription elongation, we still do not know how U1 protects against intronic PAS usage, and whether it associates with the role of U1 in splicing needs to be further investigated, given the intimate links between 3′ processing, splicing, and transcription regulation (32).

## Experimental procedures

### Cell culture and oligonucleotide/plasmid transfection

HeLa cells were cultured in Dulbecco's modified Eagle's medium supplemented with 10% fetal bovine serum. AMO transfections were performed as previously described. The sequence of the 25-mer U1 AMO is 5ʹ-GGTATCTCCCCTGCCAGGTAAGTAT-3ʹ. The sequence of control AMO is 5ʹ-CCTCTTACCTCAGTTACAATTTATA-3ʹ. AMOs were ordered from Gene Tools. Transfection of luciferase reporter plasmid pPASPORT constructs was carried out using Lipofectamine 2000 (Thermofisher) according to the user manual. Transfection of siRNAs was carried out using Lipofectamine RNAimax (Thermofisher). The siRNA target sequences are listed in Table S4.

### MS2-tagged RNA affinity purification of mRNA 3′ processing complex

The RNA affinity purification of mRNA 3′ processing complex was carried out essentially as described before (20, 29). Protein pellet was analyzed by MS to identify 3′ processing factors or by SDS-PAGE and silver staining or Western blotting analyses. Similar MS2-tagged RNA affinity coupled with Western blotting analysis was performed, as provided in Fig. S3.

### IP

Protein IPs were carried out using standard procedures recommended by antibody supplier Abcam. For RNA IPs, RNAs were extracted by Trizol reagents (Thermofisher), Northern blot analysis of U1 snRNA was performed using 5ʹ-radiolabeled DNA probes (sequence is listed in Table S4) in ultrasensitive hybridization buffer (Thermofisher). The radioactivity signals were analyzed by PhosphorImager (Typhoon FLA 7000).

### Gel shift assays

Recombinant GST-U1-70K, GST-U1A, and GST-U1C proteins were prepared from *Escherichia coli* strain BL21 (DE3) and purified with ProteinIso GST Resin (Transgen). U1 snRNA or mutant RNA was prepared by *in vitro* transcription with T7 RNA polymerase. Gel shift assays were performed following the reported protocol (30, 40).

### DNA–biotin based pull-down assay

Biotinylated DNA oligos were ordered from SYNBIO technologies (sequences are listed in Table S4). Biotinylated DNAs were first bound to the streptavidin beads and then were incubated with HeLa NE in the polyadenylation conditions for 5 min. After biotin–streptavidin binding and washing, pull-down samples were heated at 95 °C for 5 min in 1× SDS-PAGE sample loading buffer. The eluted samples were further subject to Western blotting analysis.

### *In vitro* mRNA 3ʹ processing assays

*In vitro* cleavage/polyadenylation and *in vitro* cleavage assays were performed using P^32^-radiolabeled RNA sequences and HeLa NEs following the standard protocol as described elsewhere (20, 30, 40). HeLa NEs were purchased from IPRACELL.

### Luciferase reporter assay

HeLa cells were harvested after 24 h of transfection with pPASPORT plasmids. Luciferase activity was measured using Promega Dual-Luciferase Reporter kit and Berthold Sirius detection system.

### Nuclear/cytoplasm extraction

A Nuclear/Cytosol Extraction Kit (Abcam) was used to prepare nuclear/cytoplasm proteins/RNAs for Western blotting analysis.

### Immunostaining

Immunostaining was performed using standard procedures (https://www.abcam.cn/protocols/immunocytochemistry-immunofluorescence-protocol). Primary antibodies (U1C, sc-101549; Santa Cruz) were incubated with cells overnight at 4 °C. Secondary antibodies were ordered from Beyotime Biotechnology. The fluorescence signals were detected with a Confocal Laser Scanning Microscopy (LSM880).

### Western blotting

The primary antibodies for 3′ processing factors were purchased from Bethyl Laboratories. Other primary antibodies were purchased from Abcam or Santa Cruz Biotechnology (catalog number available on request). For secondary antibodies, we used horseradish peroxidase–conjugated antimouse/rabbit (Sigma). ECL western blotting system (Thermofisher) was used to detect the signals.

### 3′-Seq, mRNA-Seq, ChIP-Seq, and iCLIP-Seq

3′-Seq was carried out using QuantSeq Rev 3ʹ mRNA sequencing library prep kit (Lexogen). Downstream sequence analysis was carried out using our recently published pipeline QuantifyPoly(A) (25).

ChIP-Seq libraries were prepared using the ChIP-IT Express Enzymatic Shearing (Active Motif) and ChIP-Seq library preparation (Vazyme) kits. Primary antibodies were purchased from Abcam or Santa Cruz Biotechnology. These libraries were sequenced on the NovaSeq platform, and all ChIP-Seq data processing and analysis were performed according to the ENCODE ChIP-Seq pipeline (https://www.encodeproject.org/data-standards/chip-seq/).

iCLIP-Seq library preparation and bioinformatics analysis were conducted as previously described (30, 41). The primary antibodies (WDR33 [A301–152A] and CstF64 [A301–092A]) were purchased from Bethyl.

## Data availability

All the deep sequencing data have been deposited to Gene Expression Omnibus database with the accession no. GSE192943. The MS proteomics data have been deposited to the ProteomeXchange Consortium with the dataset identifier PXD040405.

## Supporting information

This article contains supporting information.

## Conflict of interest

The authors declare that they have no conflicts of interest with the contents of this article.
